# Exploring dietary behaviors among healthcare providers: based on association rule mining

**DOI:** 10.3389/fpubh.2026.1726882

**Published:** 2026-02-11

**Authors:** Ting Shu, Lin xin Xie, Zi jun Yuan, Xiao qi Fan, Meng wei Jiang, De tian Liu, Jian jing Wang, Hong zhen Xie

**Affiliations:** 1 School of Nursing, Southern Medical University, Guangzhou, Guangdong, China; 2 Health Science Center, Yangtze University, Jingzhou City, China; 3 Department of Health Medicine, General Hospital of Southern Theater Command, Guangzhou, Guangdong, China; 4 School of Nursing, Guangdong Pharmaceutical University, Guangzhou, Guangdong, China

**Keywords:** association rule mining, eating behavior, health promotion, healthcare providers, influencing factors

## Abstract

**Objective:**

To identify determinants influencing dietary behaviors of healthcare providers, demonstrate the association patterns between factors, and provide scientific reference for this group to achieve and maintain healthier dietary behaviors.

**Methods:**

From July to August 2025, we conducted a cross-sectional survey targeting healthcare providers in a tertiary hospital. Univariate and multivariate analyzes were performed to identify associated factors. Subsequent association rule mining was employed to detect significant factor interaction patterns.

**Results:**

We analyzed the dietary behaviors of 1,135 healthcare providers; 81.6% were female and 61.9% were nurses. Nine influencing factors were identified, including age, educational level, and primary dining method. A total of 73 strong association rules were identified, from which experts selected 10 based on clinical knowledge and experience.

**Conclusion:**

Healthcare providers’ dietary behaviors are influenced by multi-level factors. To facilitate the adoption of healthy eating behaviors, targeted health promotion programs should be developed to address these influencing factors.

## 1 Introduction

Healthcare Providers (HCPs), including physicians, nurses, dietitians, and pharmacists, serve as frontline personnel in health promotion and disease prevention. Their dietary behaviors play a crucial role in maintaining personal health, demonstrating professional competence, and establishing credibility (1). Despite their comprehensive knowledge of healthy diets, suboptimal eating habits are frequently caused by occupational or behavioral constraints. For instance, approximately 65% of nurses in the US report poor dietary quality. In the UK, up to three-quarters of nurses consume fewer than the recommended five portions of fruits and vegetables (2). A similar situation is equally prominent in Asian countries. A survey of Chinese doctors showed that unhealthy eating habits are prevalent and account for a high proportion in this group. The proportion of eating out is increasing, with 23.0% choosing restaurants and takeout, the frequency of eating at home is decreasing, and 30.5% of doctors have irregular eating patterns (3). In South Korea, 77% of nurses have irregular eating habits, and more than half of them frequently consume fried foods, high-fat foods, and high-carbohydrate snacks (4). In Japan, the dietary structure of nurses is dominated by convenient and highly processed foods (5). In light of these findings, public health institutions and policymakers should pay attention to healthcare providers’ dietary behaviors and conditions by designing targeted strategies to improve overall health status, thereby improving their capacity to deliver quality patient care (6).

## 2 Background

With increasing research on the relationship between lifestyle and disease, maintaining healthy lifestyles has been demonstrated as the most economical approach to non-communicable disease prevention (7). Among these aspects, diet constitutes a significant part of one’s lifestyle (8). Nevertheless, healthcare workers in hospital settings frequently demonstrate suboptimal dietary patterns, inadequate physical activity, and elevated stress levels (9). This phenomenon stems firstly from their adoption of health-promoting behaviors frequently taking lower priority than patient care delivery (10); Secondly, this population works in shift schedules, particularly night shifts, which can cause circadian rhythm disruption affecting their sleep hygiene and resulting in poor dietary habits (11), exerting detrimental effects on health (3, 12). These occupationally-induced health issues require active attention from multiple sectors.

Association Rule Mining (ARM) is a process that reveals the interdependence and correlation between different factors. As an important technique in data mining, it serves as a crucial technology in data mining. It enables the extraction of valuable related data items from massive datasets and reflects the extent of their association (13). It has achieved remarkable results in clinical medicine, pharmacy and other fields (14).

Although research on healthcare providers’ occupational health is increasing, comprehensive studies examining determinants of their eating patterns are still scarce and dispersed. Current research primarily concentrates on individual occupational categories, including nurses and physicians (3, 15), or investigates determinants, including but not limited to stress, anxiety, and health literacy (16, 17). And primarily relying on traditional statistical methods, demonstrating advantages in verifying causal relationships and quantifying association strength, yet exhibit limited capacity in capturing co-occurrence patterns among variables in high-dimensional data, whereas association rules can bridge the limitations of causal analysis. Consequently, the study aims to explore the relevant factors affecting the dietary behaviors of this group from multiple dimensions, including workplace environmental characteristics, stress, individual lifestyle, and social environmental factors. Additionally, an association rule mining will be conducted to supplement the results and excavate the combined effects among multiple variables.

## 3 Materials and methods

### 3.1 Design

The study is a cross-sectional survey designed to examine the dietary behavior status of healthcare providers and analyze relevant influencing factors.

### 3.2 Setting and sample

This study employed convenience sampling from July to August 2025 to distribute questionnaires to healthcare workers at a tertiary hospital in Guangzhou to obtain relevant research data.

According to the requirement for variable-influence studies, the sample size should be at least 10–20 times the number of variables (18). The study involved 26 variables in total (See all the independent variables in Table 1), accounting for 20% invalid samples. The planned sample size ranged from 325 to 650 cases, and 1,135 subjects ultimately enrolled. To further verify the adequacy of the sample size, this study calculates the statistical power using G*Power 3.1 software (19). The effect size was set to 0.3 (20), *α* is set to 0.05, and the results show that the statistical power of this study is > 80%.

**Table 1:** Variable assignment and coding.

Type	Variable	Coding scheme
Independent variable	Sex	1 = male; 2 = female
	Age	1 = 22–26; 2 = 27–31; 3 = 32–36; 4 = ≥37
	Educational level	1 = Associate degree or below; 2 = Bachelor’s degree; 3 = Master’s degree or above
	Occupation	1 = Physician; 2 = Nurse; 3 = Pharmacist; 4 = Technician
	Department	1 = Internal Medicine; 2 = Surgery; 3 = Medical Technology/Outpatient Department; 4 = other
	Marriage	1 = unmarried; 2 = married; 3 = other
	Place of usual residence	1 = south; 2 = north
	Living situation	1 = Live alone; 2 = Live with others; 3 = Live with family; 4 = Live with spouse
	Years of work experience	1 = 0–5; 2 = 6–10; 3 = 11–15; 4 = >16
	Number of chronic diseases	1 = 0; 2 = 1; 3 = ≥2
	Take medicine	0 = no; 1 = yes
	Night shifts per month	1 = 0; 2 = <5; 3 = 5–9; 4 = >9
	Night shift pattern	1 = No night shifts; 2 = On-call duty; 3 = Rotating shifts; 4 = Permanent night shift
	work-related meal delays/skipping	1 = Occasionally; 2 = Sometimes; 3 = Often
	Smoking	0 = no; 1 = yes
	Drink alcohol	0 = no; 1 = yes
	Exercise	0 = no; 1 = yes
	Sleep quality	1 = Poor; 2 = Moderate; 3 = Good
	Perceived stress	1 = No; 2 = Mild; 3 = Moderate; 4 = Severe
	Self-rated health	1 = Poor; 2 = Moderate; 3 = Good
	Family-monitored diet	1 = Occasionally; 2 = Sometimes; 3 = Often
	Satisfaction with community/workplace dining facilities	1 = Dissatisfied; 2 = Neutral; 3 = Satisfied
	Promotion of healthy eating in communities or workplaces	1 = Occasionally; 2 = Sometimes; 3 = Often
	Primary dining method	1 = Home-cooked; 2 = Takeaway; 3 = cafeteria; 4 = Other
	Proactively consult dietary information	0 = no; 1 = yes
	Frequency of dining out	1 = Occasionally; 2 = Sometimes; 3 = Often
Dependent variable	Healthy eating behavior	0 = no; 1 = yes

### 3.3 Inclusion and exclusion criteria

Inclusion criteria: (1) Age ≥20 years; (2) Medical professionals, including but not limited to doctors, nurses, and pharmacists; (3) Existing employees; (4) Provided informed consent voluntarily. Exclusion criteria: active or uncontrolled severe organic diseases, including but not limited to severe cardiovascular and cerebrovascular diseases, liver and kidney diseases, and malignant tumors.

### 3.4 Data collection and quality control

The data collection instrument was a self-designed questionnaire tailored for this investigation, refined through literature synthesis and expert consultation. We conducted a systematic search in some databases to identify variables potentially influencing dietary behavior, and incorporated work-related factors that reflect the distinctive characteristics of healthcare providers. After the initial draft of the questionnaire was developed, it was refined under the guidance of two domain experts. It comprises seven primary sections: basic information, work-related characteristics, lifestyle, psychological cognition, social environmental factors, diet-related factors, and dietary behavior survey. Detailed variable descriptions for each section are provided in the following text. The complete English version of the questionnaire is available as Supplementary File 1.

Data collection was conducted through the institutional “Xiaodong Health” digital platform. As a nationally recognized e-mental health service provider in China, with current implementation across more than 1,000 healthcare institutions in China, which has obtained the third-level certification of Information System Security Protection, ensuring data security and confidentiality for this study. The study questionnaires were programmed by engineers to generate QR codes automatically, allowing participants to scan and complete the survey with all records securely stored in the system platform.

The questionnaire included standardized instructions, and all items were mandatory. The system was programmed to permit a single response per registered user. Questionnaires were completed following documented informed consent procedures. The completion of the questionnaires was guided by several nursing postgraduates who had received unified training. For any questionable parts, explanations were provided based on actual circumstances. After the questionnaires were completed, researchers checked them one by one. Once the questionnaires were collected, engineers assisted in exporting the data, and two nursing postgraduates independently inspected the invalid questionnaires, excluding those with regular response patterns or incomplete filling.

### 3.5 Variables and measurement

The independent variables in this study encompass multiple dimensions. Demographic characteristics include gender, age, education level, occupation, etc., totaling 11 variables. Work environment features include night shifts per month, night shift patterns, and work-related meal delays/skipping (measured by asking “Do you delay or skip meals due to work reasons?”) (21). Lifestyle factors include smoking, drinking alcohol, exercise, and sleep quality, totaling 4 variables. Smoking was defined as consuming ≥1 per day for at least 1 year (22), exercise was defined as no intentional physical exercise in the past year (23), and drinking alcohol was defined as drinking at least once per month (24). Sleep quality was measured with reference to the item “How is your sleep quality?” (25). Psychological factors include perceived stress and self-rated health status, two variables. Stress is measured by “How have you been feeling stressed in the past month?” (26), and self-rated health is measured by “How would you rate your overall health during the past year?” (25). Social environment factors include family-monitored diet, etc., three variables; dietary-related factors include primary dining methods, etc., three variables. The question settings for these two dimensions are based on clinical experience and expert opinions. Variable coding and assignment are presented in Table 1.

The dependent variable was healthy dietary behavior, using the revised Healthy Eating Behavior Evaluation Scale(EBES) for Adults developed by Wei et al. (27), assessing dietary behaviors over the past 3 months. The scale comprises eight dimensions: food choice, food preparation, snacking, social/environmental influence, healthy eating awareness, dietary preference, and special diet, with a total of 38 items.t uses a 5-point Likert scale for scoring, where 1 to 5 points represent “very inconsistent,” “inconsistent,” “uncertain,” “consistent,” and “very consistent” respectively. The total score ranges from 38 to 190 points, and higher scores indicate more optimal dietary behaviors. This study established ≥75% of the total score as the cutoff for healthy eating behaviors (28). Specifically, scores ≥128 points indicated healthy eating behaviors, while scores <128 points suggested unhealthy eating behaviors. Measurement indicated, the scale’s Cronbach’s *α* coefficient was 0.846 in this study.

### 3.6 Ethical consideration

Before study commencement, all participants were informed of the research purpose, procedures, potential risks and benefits, and verbal informed consent was obtained. Participants were informed they could unconditionally withdraw at any time. For confidentiality preservation, all personally identifiable information was removed during data collection, and each participant was assigned a code. This study was performed in accordance with the Declaration of Helsinki and has been reviewed and approved by the Ethics Committee of the General Hospital of the Southern Theatre Command of the People’s Liberation Army of China, with the ethical approval number NZLLKZ2025056, the approval date of July 17, 2025.

### 3.7 Data analysis

#### 3.7.1 Univariate and multivariate analysis

Dataset management and cleaning were conducted in Microsoft Excel 2019. Participants with incomplete records were systematically removed. All statistical procedures were executed using IBM SPSS Statistics 27.0. Descriptive data were presented as relative numbers. Univariate analysis employed chi-square tests. Significant variables from univariate analysis were included in multivariate analysis, and multivariate analysis was performed using binary logistic regression. Before incorporating variables into the regression model, the Variance Inflation Factor (VIF) test is used to assess whether there is a problem of multicollinearity among variables. If the VIF value is greater than 10, the variable is excluded. All tests were two-sided with a significance level set at *α* = 0.05.

#### 3.7.2 Association rule mining (ARM)

Using the arules package in R (Version 4.5.0), the Apriori algorithm was applied for association rule mining. The association rule mining process comprised three steps: First, identifying frequent itemsets in the dataset where itemset occurrence frequency satisfied the established minimum support criterion. Second, generating association rules from frequent itemsets. Finally, evaluating and filtering the rules based on clinical relevance and threshold values to select qualified strong association rules (13, 14).

An association rule comprises an antecedent {X} and consequent {Y}, formally denoted as X → Y. The Apriori algorithm assesses rule strength using three key metrics: support, confidence, and lift. Support refers to the probability of X and Y co-occurring, indicating rule prevalence. Confidence represents the probability of Y given X, measuring rule reliability. Lift refers to the deviation of the support parameter from what would be expected if X and Y were independent. When lift > 1, it indicates a positive correlation between X and Y. Higher lift values signify stronger associations between X and Y (13, 14).

In this study, dietary behavior was set as the consequent, while variables showing statistical significance in logistic regression were assigned as antecedents. Based on sample size considerations and literature review, the following thresholds were established for rule mining (13, 14): support ≥8.0% and confidence > 60.0%, lift > 1.2. The minimum number of antecedents was set to two. If a rule meets all the above criteria, it is classified as a strong association rule. Finally, three clinical nursing experts further screened the rules based on their expertise and clinical practice.

## 4 Result

A total of 1,183 questionnaires were collected, 10 were excluded due to missing data and 43 for patterned responses. The final analytic sample comprised 1,135 valid responses, achieving a 95.94% valid response rate.

### 4.1 Characteristics of dietary behavior scores among healthcare providers

The total dietary behavior score of healthcare providers was 117.21 ± 14.03 points with an average item score of 3.08 ± 0.37 points, indicating moderate overall levels. The food preparation dimension scored highest in the scale, healthy eating awareness was at moderate levels, while snacking and special diet dimensions scored lower. Complete data are presented in Table 2.

**Table 2:** Dietary behavior scores of healthcare providers.

Dimension	Item count	Total score ( $\bar{x}$ ± *s*)	Mean item score ( $\bar{x}$ ± *s*)
Total dietary behavior score	38	117.21 ± 14.03	3.08 ± 0.37
Food choice	3	9.05 ± 1.84	3.02 ± 0.61
Food preparation	5	17.33 ± 2.76	3.47 ± 0.56
Snacking	9	26.39 ± 5.49	2.93 ± 0.61
Social/environmental influence	5	15.66 ± 2.60	3.13 ± 0.52
Healthy eating awareness	7	21.00 ± 3.44	3.00 ± 0.49
Dietary preference	3	9.89 ± 2.25	3.30 ± 0.75
Special diet	6	17.89 ± 3.26	2.98 ± 0.54

### 4.2 Participant characteristics and univariate analysis

The study enrolled 1,135 healthcare providers, including 209 males (18.4%) and 926 females (81.6%). There were 199 physicians (17.5%),703 nurses (61.9%) and 145 technicians (12.8%). The majority were aged 27–31 years (n = 398, 35.1%). Most of them held bachelor’s degrees (*n* = 843, 74.3%). A minority resided long-term in northern regions (*n* = 64, 5.6%). The majority reported <5 monthly night shifts (n = 517, 45.6%), and the main shift pattern was day-night rotation (n = 569, 50.1%). Those without chronic diseases account for the majority (n = 947, 83.4%). There are 845 people with unhealthy eating behaviors (74.40%), and 290 people with healthy eating behaviors (25.6%). Univariate analysis revealed factors with statistical significance (see Table 3). In terms of demographics, these factors included age, educational level, occupation, department, marriage, living situation and years of work experience. In the work environment, night shifts per month, night shift pattern and work-related meal delays/skipping were all significant. In the lifestyle and psychological domains, drink alcohol, exercise, sleep quality, perceived stress, and self-rated health status showed differences among groups. Social-related factors included family-monitored diet, satisfaction with community/workplace dining facilities, promotion of healthy eating in communities or workplaces. Among diet-related factors, primary dining method, proactively consult dietary information demonstrated significant intergroup differences.

**Table 3:** Results of participant characteristic and univariate analysis.

Variables	*N* (%)	Dietary behavior		*χ* ^2^	*P*
		Unhealthy (*n* = 845,74.40%)	Health (*n* = 290,25.6%)		
Basic information					
Sex				3.463	0.063
Male	209(18.4%)	145(69.4%)	64(30.6%)		
Female	926(81.6%)	700(75.6%)	226(24.4%)		
Age				58.404	<0.001
22–26	193(17.0%)	165(85.5%)	28(14.5%)		
27–31	398(35.1%)	328(82.4%)	70(17.6%)		
32–36	235(20.7%)	164(69.8%)	71(30.2%)		
≥37	309(27.2%)	188(60.8%)	121(39.2%)		
Educational level				11.215	0.004
Associate degree or below	142(12.5%)	118(83.1%)	24(16.9%)		
Bachelor’s degree	843(74.3%)	628(74.5%)	215(25.5%)		
Master’s degree or above	150(13.2%)	99(66.0%)	51(34.0%)		
Occupation				44.916	<0.001
Physician	199(17.5%)	141(70.9%)	58(29.1%)		
Nurse	703(61.9%)	564(80.2%)	139(19.8%)		
Pharmacist	88(7.8%)	45(51.1%)	43(48.9%)		
Technician	145(12.8%)	95(65.5%)	50(34.5%)		
Department				33.795	<0.001
Internal medicine	244(21.5%)	197(80.7%)	47(19.3%)		
Surgery	338(29.8%)	272(80.5%)	66(19.5%)		
Medical technology/outpatient department	243(21.4%)	149(61.3%)	94(38.7%)		
Other	310(27.3%)	227(73.2%)	83(26.8%)		
Marriage				37.112	<0.001
Unmarried	534(47.0%)	438(82.0%)	96(18.0%)		
Married	589(51.9%)	395(67.1%)	194(32.9%)		
Other	12(1.1%)	12(100.0%)	0(0.0%)		
Place of usual residence				1.649	0.199
South	1,071(94.4%)	793(74.0%)	278(26.0%)		
North	64(5.6%)	52(81.3%)	12(18.8%)		
Living situation				40.724	<0.001
Live alone	284(25.0%)	234(82.4%)	50(17.6%)		
Live with others	217(19.1%)	185(85.3%)	32(14.7%)		
Live with family	441(38.9%)	293(66.4%)	148(33.6%)		
Live with spouse	193(17.0%)	133(68.9%)	60(31.1%)		
Years of work experience				46.672	<0.001
0–5	387(34.1%)	317(81.9%)	70(18.1%)		
6–10	313(27.6%)	244(78.0%)	69(22.0%)		
11–15	222(19.6%)	162(73.0%)	60(27.0%)		
>16	213(18.8%)	122(57.3%)	91(42.7%)		
Number of chronic diseases				1.143	0.565
0	947(83.4%)	710(75.0%)	237(25.0%)		
1	140(12.3%)	102(72.9%)	38(27.1%)		
≥2	48(4.2%)	33(68.8%)	15(31.3%)		
Take medicine				0.065	0.799
No	1,068(94.1%)	796(74.5%)	272(25.5%)		
Yes	67(5.9%)	49(73.1%)	18(26.9%)		
Work-related features					
Night shifts per month				21.805	<0.001
0	209(18.4%)	138(66.0%)	71(34.0%)		
<5	517(45.6%)	372(72.0%)	145(28.0%)		
5–9	311(27.4%)	257(82.6%)	54(17.4%)		
>9	98(8.6%)	78(79.6%)	20(20.4%)		
Night shift pattern				20.064	<0.001
No night shifts	225(19.8%)	143(63.6%)	82(36.4%)		
On-call duty	332(29.3%)	246(74.1%)	86(25.9%)		
Rotating shifts	569(50.1%)	449(78.9%)	120(21.1%)		
Permanent night shift	9(0.8%)	7(77.8%)	2(22.2%)		
Work-related meal delays/skipping				61.164	<0.001
Occasionally	355(31.3%)	219(61.7%)	136(38.3%)		
Sometimes	452(39.8%)	338(74.8%)	114(25.2%)		
Often	328(28.9%)	288(87.8%)	40(12.2%)		
Lifestyles factors					
Smoking				0.991	0.319
No	1,084(95.5%)	804(74.2%)	280(25.8%)		
Yes	51(4.5%)	41(80.4%)	10(19.6%)		
Drink alcohol				5.134	0.023
No	1,045(92.1%)	769(73.6%)	276(26.4%)		
Yes	90(7.9%)	76(84.4%)	14(15.6%)		
Exercise				34.838	<0.001
No	668(58.9%)	540(80.8%)	128(19.2%)		
Yes	467(41.1%)	305(65.3%)	162(34.7%)		
Sleep quality				36.209	<0.001
Poor	66(5.8%)	62(93.9%)	4(6.1%)		
Moderate	639(56.3%)	501(78.4%)	138(21.6%)		
Good	430(37.9%)	282(65.6%)	148(34.4%)		
Psychological factors					
Perceived stress				42.005	<0.001
No	124(10.9%)	69(55.6%)	55(44.4%)		
Mild	639(56.3%)	465(72.8%)	174(27.2%)		
Moderate	342(30.1%)	283(82.7%)	59(17.3%)		
Severe	30(2.6%)	28(93.3%)	2(6.7%)		
Self-rated health				35.858	<0.001
Poor	68(6.0%)	58(85.3%)	10(14.7%)		
Moderate	479(42.2%)	393(82.0%)	86(18.0%)		
Good	588(51.8%)	394(67.0%)	194(33.0%)		
Social factors					
Family-monitored diet				39.555	<0.001
Occasionally	204(18.0%)	170(83.3%)	34(16.7%)		
Sometimes	391(34.4%)	319(81.6%)	72(18.4%)		
Often	540(47.6%)	356(65.9%)	184(34.1%)		
Satisfaction with community/workplace dining facilities				31.014	<0.001
Dissatisfied	191(16.8%)	156(81.7%)	35(18.3%)		
Neutral	697(61.4%)	538(77.2%)	159(22.8%)		
Satisfied	247(21.8%)	151(61.1%)	96(38.9%)		
Promotion of healthy eating in communities or workplaces				7.756	0.021
Occasionally	775(68.3%)	596(76.9%)	179(23.1%)		
Sometimes	310(27.3%)	214(69.0%)	96(31.0%)		
Often	50(4.4%)	35(74.4%)	15(25.6%)		
Dietary-related factors					
Primary dining method				66.051	<0.001
Home-cooked	409(36.0%)	260(63.6%)	149(36.4%)		
Takeaway	347(30.6%)	307(88.5%)	40(11.5%)		
Cafeteria	282(24.8%)	199(70.6%)	83(29.4%)		
Other	97(8.5%)	79(81.4%)	18(18.6%)		
Proactively consult dietary information				15.738	<0.001
No	982(86.5%)	751(76.5%)	231(23.5%)		
Yes	153(13.5%)	94(61.4%)	59(38.6%)		
Frequency of dining out				4.836	0.089
Occasionally	487(42.9%)	357(73.3%)	130(26.7%)		
Sometimes	536(47.2%)	395(73.7%)	141(26.3%)		
Often	112(9.9%)	93(83.0%)	19(17.0%)		

### 4.3 Binary logistic regression

The results with statistical significance in the univariate analysis were taken as independent variables, with healthy dietary behavior as the dependent variable, and all variables had a VIF < 5, with the highest VIF value being 4.631, indicating no significant multicollinearity. Binary logistic regression analysis was performed. With the initial category of all variables designated as the reference group. Complete variable-specific outcomes are presented in Table 4. The regression analysis revealed that age, Educational level, drinking alcohol, Sleep quality, Exercise, Family-monitored diet, work-related meal delays/skipping, Primary dining method, and proactively consulting dietary information serve as independent determinants of healthy eating behaviors.

**Table 4:** Binary logistic regression analysis of the influence of various factors on dietary behavior.

Variables	Reference group	*β*	*SE*	Wald *χ* ^2^	*Ρ*	Odds ratio (95%CI)
Age	22–26					
27–31		0.208	0.326	0.408	0.523	1.232(0.650 ~ 2.335)
32–36		1.090	0.434	6.296	0.012	2.975(1.269 ~ 6.971)
≥37		0.991	0.530	3.490	0.062	2.693(0.952 ~ 7.616)
Educational level	Associate degree or below					
Bachelor’s degree		0.177	0.287	0.379	0.538	1.193(0.680 ~ 2.094)
Master’s degree or above		1.006	0.428	5.516	0.019	2.735(1.181 ~ 6.333)
Drink alcohol	No					
Yes		−0.783	0.351	4.978	0.026	0.457(0.230 ~ 0.909)
Exercise	No					
Yes		0.565	0.163	11.936	<0.001	1.759(1.277 ~ 2.424)
Sleep quality	Poor					
Moderate		1.020	0.566	3.251	0.071	2.774(0.915 ~ 8.410)
Good		1.360	0.575	5.598	0.018	3.896(1.263 ~ 12.021)
Family-monitored diet	Occasionally					
Sometimes		0.077	0.264	0.085	0.771	1.080(0.644 ~ 1.812)
Often		0.755	0.247	9.313	0.002	2.128(1.310 ~ 3.455)
Work-related meal delays/skipping	Occasionally					
Sometimes		−0.457	0.184	6.200	0.013	0.633(0.442 ~ 0.907)
Often		−0.793	0.241	10.852	<0.001	0.452(0.282 ~ 0.725)
Primary dining method	Home-cooked					
Takeaway		−0.852	0.235	13.133	<0.001	0.426(0.269 ~ 0.676)
Cafeteria		−0.087	0.203	0.186	0.666	0.916(0.616 ~ 1.363)
Other		−0.503	0.324	2.410	0.121	0.605(0.320 ~ 1.141)
Proactively consult dietary information	No					
Yes		0.445	0.222	4.015	0.045	1.560(1.010 ~ 2.412)

Omnibus test *p* < 0.01, Hosmer-Lemeshow test *p* = 0.211.

### 4.4 Association rule mining

#### 4.4.1 Frequent itemsets

Itemsets with occurrence frequency >8% were defined as frequent itemsets. After analysing all data, 1,355 frequent itemsets were generated. The top 20 itemsets ranked by support are listed in Table 5.

**Table 5:** Top 20 frequent itemsets (sorted by support).

Items	Support	Count
DA = NO	0.921	1,045
PCDI = No	0.865	982
{DA = NO, PCDI = No}	0.795	902
{EB = Unhealthy}	0.744	845
{EL = Bachelor}	0.743	843
{EL = Bachelor, DA = NO}	0.699	793
{DA = NO, EB = Unhealthy}	0.678	769
{PCDI = No, EB = Unhealthy}	0.662	751
{EL = Bachelor, PCDI = No}	0.638	724
{DA = NO, PCDI = No, EB = Unhealthy}	0.603	684
{EL = Bachelor, DA = NO, PCDI = No}	0.597	678
{Exercise = No}	0.589	668
{SQ = Moderate}	0.563	639
{EL = Bachelor, EB = Unhealthy}	0.553	628
{DA = NO, Exercise = No}	0.551	625
{Exercise = No, PCDI = No}	0.536	608
{DA = NO, SQ = Moderate}	0.517	587
{EL = Bachelor, DA = NO, EB = Unhealthy}	0.517	587
{DA = NO, Exercise = No, PCDI = No}	0.500	568
{SQ = Moderate, PCDI = No}	0.488	554

DA, Drink alcohol; PCDI, Proactively consult dietary information; EB, Eating behavior; EL, Educational level; SQ, Sleep quality.

#### 4.4.2 Rules mining

Based on the results in Table 5, the Apriori algorithm was used to mine association rules among the data, yielding 467 relevant rules. The rules had a maximum antecedent count of 7, achieving peak values of 0.677 support, 0.956 confidence, and 1.28 lift. Filtered according to strong rule criteria, a total of 73 strong rules were obtained. Finally, 10 rules were selected by combining work experience and knowledge, all with confidence >90% and support > 1.23, indicating strong predictive power of these combinations and significantly stronger positive X → Y correlations than random expectation (details in Table 6).

**Table 6:** Results of a Apriori algorithm of eating behavior.

Number	LHS	RHS	Support	Confidence	Lift	Count
1	{Age = 22–26, PDM = Takeaway}	{EB=Unhealthy}	0.086	0.951	1.278	98
2	{SQ = Moderate, Exercise = No, PCDI=No, WRMDS = Often}	{EB=Unhealthy}	0.103	0.951	1.278	117
3	{PDM = Takeaway, WRMDS = Often}	{EB=Unhealthy}	0.120	0.944	1.269	136
4	{DA = NO, SQ = Moderate, Exercise = No, WRMDS = Often}	{EB=Unhealthy}	0.100	0.942	1.265	114
5	{EL = Bachelor, PDM = Takeaway, PCDI=No, WRMDS = Often}	{EB=Unhealthy}	0.080	0.938	1.260	91
6	{PDM = Takeaway, PCDI=No, FMD = Sometimes}	{EB=Unhealthy}	0.095	0.931	1.251	108
7	{Exercise = No, PDM = Takeaway, WRMDS = Often}	{EB=Unhealthy}	0.081	0.929	1.248	92
8	{PDM = Takeaway, FMD = Sometimes}	{EB=Unhealthy}	0.107	0.924	1.241	122
9	{SQ = Moderate, PDM = Takeaway}	{EB=Unhealthy}	0.169	0.923	1.240	192
10	{Exercise = No, WRMDS = Often}	{EB=Unhealthy}	0.175	0.917	1.232	199

LHS, left hand side; RHS, right hand side; EB, Eating behavior; PDM, Primary dining method; WRMDS, work-related meal delays/skipping; DA, Drink alcohol; PCDI, proactively consult dietary information; EL, Educational level; SQ, Sleep quality; FMD, family-monitored diet.

## 5 Discussion

This study aims to identify relevant factors influencing the dietary behaviors of healthcare providers, and to recognize combined patterns among these factors through association rule mining, demonstrating the combined effects of various factors on dietary behaviors.

The results showed that age, education level, drinking alcohol, exercise, sleep quality, family-monitored diet, work-related meal delays or skips, primary dining method, and proactively consulting dietary information were associated with healthy eating behaviors. However, department, occupation, position, number and patterns of night shifts, perceived stress, and self-rated health status showed no association with healthy eating behaviors. Furthermore, given the predominant proportion of participants with unhealthy dietary patterns (*n* = 845, 74.40%) and the more concentrated clustering of factors associated with unhealthy eating, the corresponding itemsets demonstrated elevated support and confidence levels, consequently yielding a greater number of association rules about unhealthy dietary behaviors in the final analysis.

### 5.1 Healthcare providers’ dietary behaviors were at moderate levels showing a cognition-practice gap

The findings indicated that healthcare providers’ overall dietary practice scores averaged 117.21 ± 14.03, at a moderate level that still requires improvement. The average item score was 3.08 ± 0.37, consistent with the findings of Wang et al. (28) in their study of young and middle-aged populations. Among all dimension scores, food preparation had the highest average score, followed by dietary preferences, while healthy eating awareness scored moderately. The lowest scores were observed in special diet and snack dimensions, indicating that healthcare providers possess good cooking skills, nutritional knowledge and healthy eating awareness, but there is a discrepancy between their health cognition and practice, with occupational characteristics significantly impacting their dietary behaviors. Several reasons may explain these findings: Primarily, healthcare professionals receive foundational nutrition education during their training, equipping them with sound dietary awareness and essential nutritional knowledge. Moreover, a considerable proportion are aged ≥37 years, an age group that balances work and family care (29) and tends to pay more attention to the practice of healthy eating. Secondly, our investigation was conducted in Guangdong Province, where the local diet is characterized by low salt, low oil and mild spices (30), resulting in higher scores in the overall dietary preference dimension, reflecting the potential positive influence of regional dietary characteristics on health behaviors. Finally, since shift workers constituted the majority in this study, their high-intensity work led to strong dependence on snacks (31), accounting for the poor performance in snacking behavior management. Additionally, insufficient rest time during clinical work reduced the possibility of regular meals and deprived opportunities to practice healthy eating behaviors such as chewing slowly (2). Based on the above, the core contradiction of unhealthy eating behaviors among healthcare providers lies in the disconnection between cognition and practice under occupational environmental constraints. To address this, multi-level interventions are needed, including optimising healthy food provision, dietary behavior training in work settings, and supportive system design.

### 5.2 Healthcare providers’ dietary behaviors are shaped by multi-level determinants

#### 5.2.1 Demographic characteristics

Older individuals showed higher compliance rates with healthy diets, consistent with trends found in studies by Hu (32) and Krieger et al. (33). Participants aged 32–36 were 2.975 times more likely to adopt healthy eating behaviors compared to the 22–26 age group (*p* = 0.012, *OR* = 2.975). With increasing age, occupational stability and economic capacity improve; in addition, the increase in future health plans (34), and the decline in metabolic levels (35), collectively promote the adoption of healthier dietary practices. Higher education levels were associated with a greater likelihood of following healthy eating behaviors, consistent with findings from Hu (32), Zhang et al. (36). Practitioners with master’s degrees or above were 2.735 times more likely to adopt a healthy diet compared to those with associate degrees or below (*p* = 0.019, *OR* = 2.735). This disparity may stem from higher-educated individuals’ superior health literacy and greater access to health resources. In light of these findings, a targeted intervention strategy should be implemented. Focusing dietary education on younger populations while enhancing the senior staff’s role as healthy eating exemplars. For interventions targeting groups with low educational levels, it is necessary to simplify the forms and content of health information transmission and focus on diversifying the forms of dietary education, such as holding nutrition knowledge lectures and carrying out healthy diet workshops (37), to help them better understand and achieve healthy eating.

#### 5.2.2 Workplace characteristics

Work-related meal delays or skipping were negatively correlated with healthy eating behaviors, with decreasing occurrence probability at higher frequencies (*OR* = 0.633 and 0.452, *P* < 0.05), which is consistent with the trend observed by McCurley et al. (38). Owing to demanding workloads, medical providers frequently encounter delayed meal times, potentially resulting in shifted caloric intake and diminished nutritional quality, thereby contributing to glucose intolerance, dyslipidemia, and sleep disturbances (39). Consequently, multi-level intervention strategies should be developed based on the occupational characteristics of this population. Primarily, meal accessibility for medical staff must be prioritised through initiatives like unit-based meal distribution systems. Subsequently, dual approaches are recommended: optimising work schedules and shift arrangements, and creating a supportive, healthy eating environment—such as implementing nutrient labelling in hospital cafeterias and reducing the supply of high-sugar and high-salt foods (37).

#### 5.2.3 Individual lifestyle factors

Drinking alcohol showed a negative correlation with healthy dietary behaviors (*β* = −0.783, *p* = 0.026), being 0.457 times that of non-drinkers (*OR* = 0.457), which is consistent with the findings of Mei et al. (40). The underlying mechanism involves alcohol-induced enhancement of high-calorie food preference and promotion of caloric consumption (41). Furthermore, alcohol-associated eating patterns are socially contextualized, which may lead to irrational eating behaviors (42). Individuals who exercise were 1.759 times more likely to choose healthy dietary behaviors than non-exercisers (*OR* = 1.759), consistent with the study by Fernandes et al. (43). Exercise and dietary behaviors interact through shared neurocognitive mechanisms, and multiple studies indicate that individuals with higher physical activity levels tend to have healthier diets, consuming more fiber, less fat, and closer to dietary advice (44). Sleep quality was positively correlated with healthy eating behaviors (*β* = 1.360), which is consistent with the conclusion drawn by Kim et al. (45). The possible mechanism is that sleep deprivation or poor sleep quality may lead to changes in appetite-related hormones, thereby increasing appetite, especially for high-calorie and high-sugar foods; poor sleep quality can affect the function of the prefrontal cortex, reducing self-control and leading to increased selection of unhealthy foods (46). Therefore, given the intrinsic connections between various lifestyle factors, improving overall lifestyle is more meaningful than modifying a single lifestyle factor (47), to develop integrated lifestyle modification strategies specifically designed for the healthcare providers.

#### 5.2.4 Dietary-related factors

The primary eating method was negatively correlated with healthy eating behaviors (*β* = −0.852, *p* < 0.001). The incidence rate of healthy eating behaviors among those mainly consuming takeaway food was 0.426 times that of those cooking at home (OR = 0.426). Takeaway food often has insufficient content of vegetables and high-fiber grains, while containing excessive salt, sugar, and saturated fat. Moreover, consuming takeaway food reduces interaction and dietary planning during family meals (48), leading to progressive deterioration in overall dietary quality. Therefore, it is necessary to carry out targeted nutrition and health education for healthcare providers, with the primary goal of training meal preparation and storage methods suitable for shift workers, the secondary goal of improving the ability to make healthy choices in takeaway scenarios, and attention should also be paid to the construction of catering facilities in work units.

Active consultation of dietary information was positively correlated with healthy behaviors (*β* = 0.445, 0.045), and the odds were 1.56 times that of those who did not consult actively (*OR* = 1.56). Active consultation of dietary information can help individuals regain a sense of control over their own health, meet their psychological need for autonomous decision-making (49), and individuals who obtain dietary guidance through professional channels are more likely to change their dietary behaviors (50). Therefore, attention should be paid to cultivating individuals’ ability to seek health information, reducing the threshold for consultation through diversified consultation channels, enhancing the accessibility and visibility of healthy diet consultation services, and focusing on ensuring the professionalism and reliability of healthy diet information.

#### 5.2.5 Social environmental factors

The analysis revealed significantly higher odds of healthy eating patterns among participants with regular family dietary monitoring compared to occasional monitoring (*OR* = 2.218) (2). Possible reasons include that family monitoring can influence individual food choices through reminders, encouragement, or joint participation. Regular attention may create a positive supervision mechanism that reinforces healthy eating behaviors. The results suggest that dietary interventions should emphasize the potential role of family members by enhancing family health awareness, improving home food environments, and strengthening positive interactions to establish a family ecosystem supporting healthy eating.

### 5.3 Results of association rules have expanded new perspectives for guiding healthcare providers in a healthy diet

Results from association rule mining have revealed combinations of factors strongly associated with unhealthy dietary behaviors, expanding new perspectives for understanding factors related to dietary behaviors. The results indicate that work-related, social environmental, and diet-related factors interact and correlate with each other, collectively influencing unhealthy dietary behaviors.

First, takeaway is a core factor promoting unhealthy diets, appearing in all high-confidence rules (Confidence >0.9). This aligns with previous studies on nutritional imbalance in takeout food (51). Its co-occurrence with work-related meal or skipping is frequent and strongly predicts unhealthy eating (Rule 3: Support = 12%, Confidence = 0.944), consistent with Oostenbach et al. (52). Consequently, singular approaches like takeout restriction may have limited effects, requiring comprehensive interventions tailored to healthcare providers’ occupational characteristics. Additionally, young populations typically exhibit frequent takeaway consumption (Rule 1), consistent with Hu et al. (53), suggesting interventions should primarily target eating patterns for this demographic. Fatigue caused by poor sleep quality exacerbates reliance on takeaway food, and this is a common phenomenon among healthcare providers (with a confidence of 16.9% in Rule 9), suggesting that individuals with such characteristics are key targets for intervention. For the phenomenon of unhealthy diets caused by takeaway food, it is necessary not only to guide individuals to establish autonomous and self-disciplined healthy eating behaviors but also to rely on institutional nudges at the policy level—such as promoting the “traffic light” labeling system for takeaway food, which intuitively marks the content levels of sodium, sugar, and unhealthy fats in meals through green, yellow, and red labels (54).

Secondly, frequent work-related meal delays or skipping is another common factor that aligns with the occupational characteristics of healthcare workers, further supporting the results of multivariate analysis, and is often accompanied by lack of exercise and poor sleep quality (Rules 2, 4, 10). This phenomenon supports the “time poverty theory,” which prompts individuals to choose unhealthy lifestyles, such as increasing the possibility of unhealthy diets, causing sleep quality problems, and restricting exercise participation (55, 56). Attention should be paid to populations with concurrent related traits, as they have a high likelihood of developing unhealthy diets (confidence = 95.1 and 94.2%).

Thirdly, it is noteworthy that groups with occasional family dietary monitoring have higher risks of unhealthy eating, contrasting with the finding that consistent family monitoring improves healthy eating probability (*OR* = 2.128 as mentioned previously), suggesting family interventions should emphasize frequency and methods. When family monitoring is fragmented, it neither provides stable emotional support nor effectively supervises takeout substitution behaviors, often accompanying takeout dependence (Rule 8 Support = 0.107). Strengthening guidance and interventions on healthy family lifestyles has been incorporated into national health strategies (57). Healthy diet education should be conducted on a family basis, actively creating healthier home food environments, and enhancing the continuity of family attention to promote healthy eating (58).

Fourthly, Rule 5 reveals the critical role of active information acquisition. Even among individuals with higher education (EL = Bachelor), without professional nutritional counselling, the probability of unhealthy eating habits is significantly high (Confidence = 93.8%) due to frequent work-related stress and reliance on takeout food. Therefore, implementing effective dietary counselling interventions for this population is crucial, and integrating such services into workplace settings should be considered.

## 6 Conclusion

This study explored the related factors of dietary behaviors among healthcare providers, including physicians, nurses, technicians, and pharmacists. The results showed that alcohol consumption, exercise, sleep quality, family-monitored diet, work-related meal delays/skipping, primary dining method, and proactively consulting dietary information were associated with healthy dietary behaviors. In addition, association rule mining was used to further reveal the occurrence probability of unhealthy dietary behaviors under different combinations of factors. The combination of these two methods provides multi-dimensional evidence support for an in-depth understanding of the influencing mechanisms of dietary behaviors. Therefore, future health intervention initiatives should prioritise the following key dimensions: Primary interventions should target individual dietary behaviors as the entry point, prioritising sleep-deprived individuals and younger populations through strategies that enhance self-regulation of health behaviors. Secondary measures require institutional support systems tailored to healthcare providers’ work patterns, implementing integrated interventions encompassing nutritional adequacy, exercise promotion, sleep hygiene, and alcohol moderation. Tertiary strategies should activate family support networks and optimise the accessibility and clinical utility of evidence-based nutrition counselling services. This multi-level, integrated approach demonstrates strong potential for sustainably improving dietary practices in medical service populations.

## 7 Limitation

The study had some limitations. First, as a cross-sectional study, this research is inherently limited to identifying potential associations rather than establishing causal relationships. Furthermore, while association rule mining reveals co-occurrence patterns among variables, it does not provide evidence for causal inference. Future research should conduct longitudinal studies to clarify causal relationships between variables. Second, the current analysis failed to account for confounding factors such as economic income and health literacy. In addition, this study did not include specific shift work factors (such as shift frequency and shift duration) in the analysis, which is a limitation in fully understanding the dietary behaviors of healthcare service providers. Future studies could conduct in-depth exploration by refining the measurement indicators of shift variables and conduct more comprehensive investigations targeting specific populations or regions. Third, when investigating dietary-related factors, the past 3 months were used as the reference period. The advantage of this time is that it can capture relatively stable behavioral patterns of individuals, but it may not reflect shorter-term changes. Future studies could consider using tools with different time scales to more comprehensively understand dietary habit dynamics. Fourth, the single-institution sampling frame limits population representativeness relative to multi-center studies, requiring caution when generalising the conclusions.

## Data Availability

The raw data supporting the conclusions of this article will be made available by the authors, without undue reservation.

## References

[1] Plyassovskaya S Mkhitaryan X Pozdnyakova Y. Nutritional status of healthcare professionals in primary health and social care. PLoS One. (2025) 20. doi: 10.1371/journal.pone.0325422, 40465786 PMC12136426

[2] Marko S Wylie S Utter J. Enablers and barriers to healthy eating among hospital nurses: a systematic review. Int J Nurs Stud. (2023) 138:104412. doi: 10.1016/j.ijnurstu.2022.104412, 36528912

[3] Chen M Xu X Liu Y Yao Y Zhang P Liu J . Association of eating habits with health perception and diseases among Chinese physicians: a cross-sectional study. Front Nutr. (2023) 10:1226672. doi: 10.3389/fnut.2023.1226672, 37637951 PMC10452877

[4] Han K Choi-Kwon S Kim KS. Poor dietary behaviors among hospital nurses in Seoul, South Korea. Appl Nurs Res. (2016) 30:38–44. doi: 10.1016/j.apnr.2015.10.009, 27091251

[5] Okami Y Ideno Y Kishi M Miyazaki Y Nagai K Kurabayashi T . Japanese female nurses’ characteristics and diet quality associated with their food intake patterns: the Japan nurses’ health study. Int J Gastron Food Sci. (2025) 41:101236. doi: 10.1016/j.ijgfs.2025.101236

[6] Migdanis A Tsolis K Migdanis I Kaltsa AG Fytsilis FA Manouras A . The effect of shift working on dietary patterns of healthcare practitioners during the COVID-19 pandemic: a cross-sectional study. Medicina. (2024) 60:627. doi: 10.3390/medicina60040627, 38674273 PMC11051816

[7] Zhang YB Pan XF Chen J Cao A Xia L Zhang Y . Combined lifestyle factors, all-cause mortality and cardiovascular disease: a systematic review and meta-analysis of prospective cohort studies. J Epidemiol Community Health. (2021) 75:92–9. doi: 10.1136/jech-2020-214050, 32892156

[8] Crowley J Ball L Hiddink GJ. Nutrition in medical education: a systematic review. Lancet Planet Health. (2019) 3:e379–89. doi: 10.1016/s2542-5196(19)30171-8, 31538623

[9] Worley V Fraser P Allender S Bolton KA. Describing workplace interventions aimed to improve health of staff in hospital settings - a systematic review. BMC Health Serv Res. (2022) 22. doi: 10.1186/s12913-021-07418-9 PMC8991835 35392894

[10] Znyk M Kaleta D. Unhealthy eating habits and determinants of diet quality in primary healthcare professionals in Poland: a cross-sectional study. Nutrients. (2024) 16:3367. doi: 10.3390/nu16193367, 39408334 PMC11478428

[11] van Elk F Robroek SJW Smits-de Boer S Kouwenhoven-Pasmooij TA Burdorf A Oude Hengel KM. Study design of PerfectFit@night, a workplace health promotion program to improve sleep, fatigue, and recovery of night shift workers in the healthcare sector. BMC Public Health. (2022) 22:779. doi: 10.1186/s12889-022-13206-9, 35436871 PMC9014783

[12] Ling PL Lai ZY Cheng HL Lo K-H. Barriers and facilitators to healthy eating for shift-work-registered nurses in Hong Kong public hospitals: an exploratory multi-method study. Nutrients. (2025) 17. doi: 10.3390/nu17071162, 40218920 PMC11990088

[13] Han S Mo G Gao T Sun Q Liu H Zhang M. Age, sex, residence, and region-specific differences in prevalence and patterns of multimorbidity among older Chinese: evidence from Chinese Longitudinal Healthy Longevity Survey. BMC Public Health. (2022) 22:1116. doi: 10.1186/s12889-022-13506-0, 35658851 PMC9166487

[14] Li X Guo X Wang N Jia P Zhu K Huang X. Association rules analysis of diabetic distress in elderly patients with type 2 diabetes mellitus in the community. Chin Nurs Res. (2024) 38:4152–8. doi: 10.12102/j.issn.1009-6493.2024.23.003

[15] Onofrei LM Puiu M Chirita-Emandi A Serban CL. A comprehensive analysis concerning eating behavior associated with chronic diseases among Romanian community nurses. Front Public Health. (2024) 12:1368069. doi: 10.3389/fpubh.2024.1368069, 38577280 PMC10991806

[16] Celik Erden S Karakus Yilmaz B Kozaci N Uygur AB Yigit Y Karakus K . The relationship between depression, anxiety, and stress levels and eating behavior in emergency service workers. Cureus. (2023) 15:e35504. doi: 10.7759/cureus.35504, 37007378 PMC10058381

[17] Jürgensen IN Koch P Otto R Nock AM Petersen-Ewert C. Subjective health status, health-related behavior, and health literacy of health professional students: results from a cross-sectional study. Health Care (Don Mills). (2024) 12:277. doi: 10.3390/healthcare12020277, 38275556 PMC10815007

[18] Memon MA Ting H Cheah JH Thurasamy R Chuah F Cham TH. Sample size for survey research: review and recommendations. J Appl Struct Equ Model. (2020) 4:i–xx. doi: 10.47263/JASEM.4(2)01

[19] Faul F Erdfelder E Lang AG Buchner A. G*power 3: a flexible statistical power analysis program for the social, behavioral, and biomedical sciences. Behav Res Methods. (2007) 39:175–91. doi: 10.3758/bf03193146, 17695343

[20] Cohen J. A power primer. Psychol Bull. (1992) 112:155–9. doi: 10.1037/0033-2909.112.1.155, 19565683

[21] Phoi YY Rogers M Bonham MP Dorrian J Coates AM. A scoping review of chronotype and temporal patterns of eating of adults: tools used, findings, and future directions. Nutr Res Rev. (2022) 35:112–35. doi: 10.1017/S0954422421000123, 33988113

[22] Fang G Wen L Wang X Feng S Zhou Y Chen S . Association between neutrophil to high-density lipoprotein cholesterol ratio and incidence of cardiovascular disease in patients with metabolic associated fatty liver disease. Chin Circ J. (2025) 40:605–10. doi: 10.3969/j.issn.1000-3614.2025.06.008

[23] Li D Niu Z Wang H. A retrospective cohort study on health examination of elderly population in Huangpu District, Guangzhou. Chin Gen Pract. (2025) 28:1635–41. doi: 10.12114/j.issn.1007-9572.2024.0359

[24] Xu T He Y Cai Z Li L Wu Y Zhou H . Relationship between physical activity and depressive symptoms among middle-aged and older adults in Chengdu, Sichuan, China. Acta Acad Med Sin. (2024) 46:497–506. doi: 10.3881/j.issn.1000-503X.15929 39223014

[25] Chinese Health Management Association, the Editorial Board of Chinese Journal of Health Management. Expert consensus on basic items of healthy checkup (2022). Chin J Health Manag. (2023) 17:649–60. doi: 10.3760/cma.j.cn115624-20230628-00395

[26] Li H Meng J Sun J Feng J Qiu L Yan J. Prevalence of depressive symptoms and related factors among urban residents under modern lifestyles: a cross-sectional study in Central China. Asian J Psychiatr. (2023) 86:103682. doi: 10.1016/j.ajp.2023.103682, 37385216

[27] Wei S Nie S Zhu G Lu Z. Reliability and validity of adult eating behavior evaluation questionnaire. Med Soc. (2006) 7:20–3.

[28] Wang J Gong Y Zhang Q Feng L Li Y. Relationship between healthy eating behavior among young and middle—aged people and risk of gallstone disease. J Nurs Sci. (2022) 37:6–10. doi: 10.3870/i.issn.1001—4152.2022.10.006

[29] Cecconi C Adams R Cardone A Declaye J Silva M Vanlerberghe T . Generational differences in healthcare: the role of technology in the path forward. Front Public Health. (2025) 13:1546317. doi: 10.3389/fpubh.2025.1546317, 40078753 PMC11897013

[30] Li H Jia P Fei T. Associations between taste preferences and chronic diseases: a population-based exploratory study in China. Public Health Nutr. (2021) 24:2021–32. doi: 10.1017/S136898002000035X, 32515723 PMC10195596

[31] Sajwani AI Hashi F Abdelghany E Alomari A Alananzeh I. Workplace barriers and facilitators to nurses’ healthy eating behaviours: a qualitative systematic review. Contemp Nurse. (2024) 60:270–99. doi: 10.1080/10376178.2024.2354336, 38805602

[32] Hu A Zhang X Wu Y Gao Z Tang Y Li X. Adherence to healthy lifestyles in middle-aged population in four types of communities. Chin Circ J. (2022) 37:513–8. doi: 10.3969/j.issn.1000-3614.2022.05.011

[33] Krieger JP Pestoni G Cabaset S Brombach C Sych J Schader C . Dietary patterns and their sociodemographic and lifestyle determinants in Switzerland: results from the national nutrition survey menuCH. Nutrients. (2018) 11:62. doi: 10.3390/nu11010062 30597962 PMC6356790

[34] Deeks A Lombard C Michelmore J Teede H. The effects of gender and age on health related behaviors. BMC Public Health. (2009) 9:213. doi: 10.1186/1471-2458-9-213, 19563685 PMC2713232

[35] Zampino M AlGhatrif M Kuo PL Simonsick E Ferrucci L. Longitudinal changes in resting metabolic rates with aging are accelerated by diseases. Nutrients. (2020) 12:3061. doi: 10.3390/nu12103061, 33036360 PMC7600750

[36] Zhang X Lu J Wu C Cui J Wu Y Hu A . Healthy lifestyle behaviours and all-cause and cardiovascular mortality among 0.9 million Chinese adults. Int J Behav Nutr Phys Act. (2021) 18:162. doi: 10.1186/s12966-021-01234-4, 34922591 PMC8684211

[37] Panchbhaya A Baldwin C Gibson R. Improving the dietary intake of health care workers through workplace dietary interventions: a systematic review and meta-analysis. Adv Nutr. (2021) 13:595–620. doi: 10.1093/advances/nmab120 PMC8970821 34591091

[38] McCurley JL Levy DE Dashti HS Gelsomin E Anderso E Sonnenblick R . Association of employees’ meal skipping patterns with workplace food purchases, dietary quality, and cardiometabolic risk: a secondary analysis from the ChooseWell 365 Trial. J Acad Nutr Diet. (2022) 122:110–120.e2. doi: 10.1016/j.jand.2021.08.109, 34478879 PMC9115715

[39] Dashti HS Scheer FAJL Saxena R Garaulet M. Timing of food intake: identifying contributing factors to design effective interventions. Adv Nutr. (2019) 10:606–20. doi: 10.1093/advances/nmy131, 31046092 PMC6628856

[40] Mei D Deng Y Li Q Lin Z Jiang H Zhang J . Current status and influencing factors of eating behavior in residents at the age of 18~60: a cross-sectional study in China. Nutrients. (2022) 14:2585. doi: 10.3390/nu14132585, 35807764 PMC9268282

[41] Kwok A Dordevic AL Paton G Page MJ Truby H. Effect of alcohol consumption on food energy intake: a systematic review and meta-analysis. Br J Nutr. (2019) 121:481–95. doi: 10.1017/s0007114518003677, 30630543

[42] Fong M Scott S Albani V Adamson A Kaner E. ‘Joining the dots’: individual, sociocultural and environmental links between alcohol consumption, dietary intake and body weight—a narrative review. Nutrients. (2021) 13:2927. doi: 10.3390/nu13092927, 34578805 PMC8472815

[43] Fernandes V Rodrigues F Jacinto M Teixeira D Cid L Antunes R . How does the level of physical activity influence eating behavior? A self-determination theory approach. Life. (2023) 13:298. doi: 10.3390/life13020298, 36836655 PMC9961293

[44] Joseph RJ Alonso-Alonso M Bond DS Pascual-Leone A Blackburn GL. The neurocognitive connection between physical activity and eating behaviour. Obes Rev. (2011) 12:800–12. doi: 10.1111/j.1467-789X.2011.00893.x, 21676151 PMC3535467

[45] Kim O Jung H. Prediction model for abnormal eating behaviour among hospital nurses: a structural equation modelling approach. Int J Nurs Pract. (2021) 27:e13006. doi: 10.1111/ijn.13006, 34363295

[46] Min C Kim HJ Park IS Park B Kim JH Sim S . The association between sleep duration, sleep quality, and food consumption in adolescents: a cross-sectional study using the Korea youth risk behavior web-based survey. BMJ Open. (2018) 8:e022848. doi: 10.1136/bmjopen-2018-022848, 30042149 PMC6059317

[47] Wu J Feng Y Zhao Y Guo Z Liu R Zeng X . Lifestyle behaviors and risk of cardiovascular disease and prognosis among individuals with cardiovascular disease: a systematic review and meta-analysis of 71 prospective cohort studies. Int J Behav Nutr Phys Act. (2024) 21:42. doi: 10.1186/s12966-024-01586-7, 38650004 PMC11036700

[48] Liu B Zhou K Lei A Wang Y Wang L. Exploring factors influencing online food consumption using multi-source data: a case study of Hongkou District, Shanghai. Urban Plan Int. (2024) 39:27–35. doi: 10.19830/j.upi.2024.373

[49] Vrinten J Van Royen K Pabian S De Backer C Matthys C. Motivations for nutrition information-seeking behavior among Belgian adults: a qualitative study. BMC Public Health. (2022) 22:2432. doi: 10.1186/s12889-022-14851-w, 36575414 PMC9792929

[50] Ruani MA Reiss MJ Kalea AZ. Diet-nutrition information seeking, source trustworthiness, and eating behavior changes: an international web-based survey. Nutrients. (2023) 15:4515. doi: 10.3390/nu15214515, 37960169 PMC10649819

[51] Rosenheck R. Fast food consumption and increased caloric intake: a systematic review of a trajectory towards weight gain and obesity risk. Obes Rev. (2008) 9:535–47. doi: 10.1111/j.1467-789X.2008.00477.x, 18346099

[52] Oostenbach LH Lamb KE Crawford D Thornton L. Influence of work hours and commute time on food practices: a longitudinal analysis of the household, income and labour dynamics in Australia survey. BMJ Open. (2022) 12:e056212. doi: 10.1136/bmjopen-2021-056212 PMC9083384 35523493

[53] Hu C Zhang M Zhang X Zhao Z Huang Z Li C . Relationship between eating behavior and obesity among Chinese adults. Chin J Epidemiol. (2020) 41:1296–302. doi: 10.3760/cma.j.cn112338-20191225-00915 32867439

[54] Gorski MT Roberto CA. Public health policies to encourage healthy eating habits: recent perspectives. J Healthc Leadersh. (2015) 7:81. doi: 10.2147/JHL.S69188, 29355201 PMC5740998

[55] Sun X Yang S Kong X Liu Z Ma R Yuan Y . Conceptualization of time poverty and its impact on well-being: from the perspective of scarcity theory. Adv Psychol Sci. (2024) 32:27–38. doi: 10.3724/sp.j.1042.2024.00027

[56] Urakawa K Wang W Alam M. Empirical analysis of time poverty and health-related activities in Japan. J Fam Econ Issues. (2020) 41:520–9. doi: 10.1007/s10834-020-09671-2

[57] State Council Opinions of the State Council on Implementing the Healthy China Initiative: State Council Document No.13 (2019) [A] (2019)

[58] Kegler MC Howard D Bundy L Owolabi S Hartman T Collins T . Impact and cost-effectiveness of a home food environment intervention on healthy eating. Am J Prev Med. (2025) 68:1130–41. doi: 10.1016/j.amepre.2025.02.017, 40054706

