# Correction: Exploring dietary behaviors among healthcare providers: based on association rule mining

**DOI:** 10.3389/fpubh.2026.1810843

**Published:** 2026-02-17

**Authors:** Frontiers Production Office

**Affiliations:** Frontiers Media SA, Lausanne, Switzerland

**Keywords:** association rule mining, eating behavior, health promotion, healthcare providers, influencing factors

Affiliation 2 “Health Science Center, Yangtze University, Jingzhou City, China” was erroneously given as “Department of Health Medicine, General Hospital of Southern Theater Command, Jingzhou, Hubei, China”.

The original version of this article has been updated.

