# Supplementary material for: Exploring dietary behaviors among healthcare providers: based on association rule mining
Source: Front Public Health. 2026 Feb 11;14:1726882. doi: 10.3389/fpubh.2026.1726882 (PMC12954736; doi:10.3389/fpubh.2026.1726882)
Supplement: Supplementary file 1 [file Table_1.DOCX]

**Questionnaire on Dietary Behavior of healthcare providers**

Dear Sir/Madam,

Hello! Healthy eating is crucial for preventing diseases and improving quality of life. This questionnaire aims to understand your dietary behaviors, collect necessary information, and enhance service quality. We sincerely invite you to participate in this survey. All information you provide will be strictly confidential and used only for this quality improvement. Please fill out the following questionnaire truthfully based on your actual situation. This questionnaire includes seven parts: personal information, lifestyle habits, work-related characteristics, etc., with a total of 65 questions. It is expected to take about 5-10 minutes to complete. We wish you a pleasant life!

Thanks for your cooperation!

**Section 1: Basic information**

**1、Sex：**

☐Male ☐Female

**2、Age：**

**3、Your educational level is:**

☐Associate degree or below ☐Bachelor's degree ☐Master's degree or above

**4、What is your occupation?**

☐Physician ☐Nurse ☐Pharmacist ☐Technician

**5、Which department are you in?**

☐Internal Medicine ☐Surgery ☐ Medical Technology ☐ Specialty

☐Outpatient Department ☐ Emergency & Critical Care ☐Management

☐ Surgical Anaesthesia

**6、What is your marital status?**

☐unmarried ☐ married ☐divorced ☐ other

**7、What is your usual place of residence? (divided by the Yangtze River)**

☐ South ☐ North

**8、What is your long-term living situation?**

☐ Live alone ☐ Live with others ☐Live with family

☐Live with spouse ☐Other

**9、How many years have you worked so far?**

☐0-5 ☐6-10 ☐10-15 ☐＞16

**10、How many of the following chronic conditions have you been diagnosed with?** (e.g., diabetes, hypertension, dyslipidemia, heart disease, renal dysfunction)

**11、Have you taken any medication in the past three months?**

☐No ☐Yes

**Section 2: Work-related features**

**1、Night shift or duty situation in the past month**：

☐ None ☐1-2 times/month ☐3-4 times/month  
☐1-2 times/week ☐3-4 times/week ☐5 times or more

**2、What is your night shift pattern?**

☐No night shifts ☐On-call duty ☐Rotating shifts

☐Permanent night shift

**3、Do you delay or skip meals due to work reasons?**

☐ Occasionally ☐Sometimes ☐ Often

**Section 3: Your Lifestyle**

**1、Are you a current smoker? (≥1 cigarette daily for ≥1 year)**

☐No ☐Yes

**2、Do you drink alcohol? (At least once per month)**

☐No ☐Yes

**3、Have you engaged in any conscious physical exercise in the past year?**

☐No ☐Yes

**4、Your sleep quality：**

☐Poor ☐Moderate ☐ Good

**Section 4: stress and self-rated health**

**1、How have you been feeling stressed in the past month?**

☐ No ☐Mild ☐ Moderate ☐ Severe

**2、How would you rate your overall health during the past year?**

☐Poor ☐Moderate ☐ Good

**Section 5: Socio-environmental part**

**1、Do your family members monitor whether your diet is healthy?**

☐ Occasionally ☐Sometimes ☐ Often

**2、Are you satisfied with the variety, price, and taste of food provided by the dining facilities in your community or workplace?**

☐ Dissatisfied ☐ Neutral ☐ satisfied

**3、Has your community or workplace conducted healthy eating promotion activities?(e.g., providing measured salt/oil containers, tape measures; delivering nutrition education lectures or events)**

☐ Occasionally ☐Sometimes ☐ Often

**Section 6:** **Dietary-related part**

Note: The items in this section are to be filled out based on your situation over the past three months.

**1、What is your primary meal pattern ?**

☐Home-cooked ☐ Takeaway ☐cafeteria ☐Other

**2、Have you proactively sought dietary information during the last three months?**

☐No ☐Yes

**3、What is your frequency of dining out?**

☐ Occasionally ☐Sometimes ☐ Often

**Section 7:** **Healthy Eating Behavior Evaluation Scale (EBES)**

The following are some descriptions of your usual dietary habits and attitudes. Please answer whether these descriptions match your personal situation based on your own circumstances. Please note that there is no right or wrong answer; you only need to choose the option that best fits your personal situation.

| Food choice | | very inconsistent | inconsistent | uncertain | consistent | very consistent |
| --- | --- | --- | --- | --- | --- | --- |
| 1 | I pay attention to the combination of different foods when eating. |  |  |  |  |  |
| 2 | I choose food mainly based on the principle of convenience. |  |  |  |  |  |
| 3 | I usually eat just a few favourite dishes and rarely vary them. |  |  |  |  |  |
| Food preparation | | very inconsistent | inconsistent | uncertain | consistent | very consistent |
| 4 | I frequently consume reheated leftover meals. |  |  |  |  |  |
| 5 | I enjoy cooking. |  |  |  |  |  |
| 6 | Taste is my primary consideration rather than health when selecting foods. |  |  |  |  |  |
| 7 | As long as I don't have to cook, I'll eat anything available. |  |  |  |  |  |
| 8 | I still eat food past its expiration date if it seems fine. |  |  |  |  |  |
| Snacking | | very inconsistent | inconsistent | uncertain | consistent | very consistent |
| 9 | When I feel bored, I eat some snacks. |  |  |  |  |  |
| 10 | I often eat snacks. |  |  |  |  |  |
| 11 | When I feel hungry before a meal, I eat some snacks to fill my stomach. |  |  |  |  |  |
| 12 | I often eat sweet snacks. |  |  |  |  |  |
| 13 | If I am very busy with work, I replace meals with snacks. |  |  |  |  |  |
| 14 | I never miss any of the three main meals. |  |  |  |  |  |
| 15 | I rarely eat snacks. |  |  |  |  |  |
| 16 | I enjoy going to supermarkets or stores to browse and buy snacks. |  |  |  |  |  |
| 17 | Sometimes I eat snacks even when I'm not hungry. |  |  |  |  |  |
| Social/environmental influence | | very inconsistent | inconsistent | uncertain | consistent | very consistent |
| 18 | When I'm in a bad mood, I don't want to eat anything. |  |  |  |  |  |
| 19 | Whether I'm in a good or bad mood, it doesn't affect my eating. |  |  |  |  |  |
| 20 | After seeing related promotions, I stopped eating foods I usually enjoy for health reasons. |  |  |  |  |  |
| 21 | I eat at home because it's cleaner than eating out. |  |  |  |  |  |
| 22 | My emotional state affects what and how much I eat. |  |  |  |  |  |
| Healthy eating awareness | | very inconsistent | inconsistent | uncertain | consistent | very consistent |
| 23 | I often take vitamin or calcium tablets. |  |  |  |  |  |
| 24 | I usually eat until about 80% full at each meal. |  |  |  |  |  |
| 25 | I choose to eat some healthy foods even though I don't like them. |  |  |  |  |  |
| 26 | I pay relatively more attention to information about healthy eating. |  |  |  |  |  |
| 27 | I often eat fried or high-fat foods. |  |  |  |  |  |
| 28 | For health reasons, my taste preferences have become lighter than before. |  |  |  |  |  |
| 29 | Even though I know some foods are unhealthy, I still eat them if they taste good. |  |  |  |  |  |
| Dietary preference | |  |  |  |  |  |
| 30 | I often drink sugary beverages. |  |  |  |  |  |
| 31 | I often eat pickled foods. |  |  |  |  |  |
| 32 | My taste preferences are relatively strong. |  |  |  |  |  |
| Special diet | |  |  |  |  |  |
| 33 | My breakfast is very irregular; sometimes I eat, sometimes I don't. |  |  |  |  |  |
| 34 | I eat very quickly. |  |  |  |  |  |
| 35 | When eating, I like to chew slowly and thoroughly. |  |  |  |  |  |
| 36 | My three daily meals are very regular. |  |  |  |  |  |
| 37 | I like to watch TV or read newspapers/books while eating. |  |  |  |  |  |
| 38 | Generally speaking, my dinner is the best and most substantial. |  |  |  |  |  |
